# A novel NIH research grant recommender using BERT

**DOI:** 10.1371/journal.pone.0278636

**Published:** 2023-01-17

**Authors:** Jie Zhu, Braja Gopal Patra, Hulin Wu, Ashraf Yaseen

**Affiliations:** 1 Department of Biostatistics and Data Science, School of Public Health, The University of Texas Health Science Center, Houston, Texas, United States of America; 2 Department of Population Health Sciences, Weill Cornell Medicine, Cornell University, New York, New York, United States of America; Torrens University Australia, AUSTRALIA

## Abstract

Research grants are important for researchers to sustain a good position in academia. There are many grant opportunities available from different funding agencies. However, finding relevant grant announcements is challenging and time-consuming for researchers. To resolve the problem, we proposed a grant announcements recommendation system for the National Institute of Health (NIH) grants using researchers’ publications. We formulated the recommendation as a classification problem and proposed a recommender using state-of-the-art deep learning techniques: i.e. Bidirectional Encoder Representations from Transformers (BERT), to capture intrinsic, non-linear relationship between researchers’ publications and grants announcements. Internal and external evaluations were conducted to assess the system’s usefulness. During internal evaluations, the grant citations were used to establish grant-publication ground truth, and results were evaluated against Recall@k, Precision@k, Mean reciprocal rank (MRR) and Area under the Receiver Operating Characteristic curve (ROC-AUC). During external evaluations, researchers’ publications were clustered using Dirichlet Process Mixture Model (DPMM), recommended grants by our model were then aggregated per cluster through Recency Weight, and finally researchers were invited to provide ratings to recommendations to calculate Precision@k. For comparison, baseline recommenders using Okapi Best Matching (BM25), Term-Frequency Inverse Document Frequency (TF-IDF), doc2vec, and Naïve Bayes (NB) were also developed. Both internal and external evaluations (all metrics) revealed favorable performances of our proposed BERT-based recommender.

## Introduction

The importance of recommendation systems can be understood from its daily usage in recommending movies, books, videos, news, products, and so on. The working of a typical recommender depends on analytic modeling of a user’s behavior based on the past preferences/statistics. It can be broadly grouped as content-based, collaborative filtering, and hybrid [1]. Given their useful applications in several areas, extending the application of recommenders to include scholarly resources, such as recommending grants for researchers, would be beneficial.

Acquisition of research grants is important for researchers to conduct research in academia. There are several funding opportunities available for researchers to help innovate and implement bright ideas. These funding opportunities are normally from different government and private sources such as NIH, National Science Foundation, Microsoft, and many more. However, searching for relevant grant announcements in a large database is always a difficult and exhausting process for researchers.

There is currently a commercial website named SPIN [2] that lists all the grants available in the USA. However, manual searches in SPIN revealed that the performance of the implemented search engine is quite poor since it can only handle very limited queries, and is only useful when the researchers know exactly what they are looking for. To the best of our knowledge, research dedicated to recommending research grant opportunities to help alleviate the problem is very limited. We were able to find only two [3,4] that were restricted to using keywords and association rules for grants opportunities in Japan, and a recent one [5] based on TF-IDF with Random forest and Rocchio algorithm. But we did find studies for other scholarly resources such as literature [6–9], collaborators [10–13] and datasets [14,15] that utilized deep learning techniques such as transformers.

Considering the research gap and outstanding performances of deep learning models on other academic recommendation tasks such as citation/paper, dataset recommendations, we proposed a novel research grant recommender based on state-of-the-art BERT model. The main contributions of our work in this area are:

- We are the first to introduce a grant recommender that utilizes the advanced, state-of-the-art natural language model, i.e. BERT, to capture intrinsic, non-linear relationship between researchers and grant opportunities.
- Complementary to our main model architecture, we additionally introduced DPMM clustering algorithm with Recency Weight for aggregation for practical applications/service purpose.
- We crawled data suitable for real-world applications: publications from the PubMed, and NIH grant opportunities from grants.gov, and the current web-based application for our recommender is available at http://genestudy.org/recommends/#/grants, giving our research a practical use. This also allowed us to collect feedback/ratings from end-users to conduct an external evaluation of the system.

**Fig 1:**
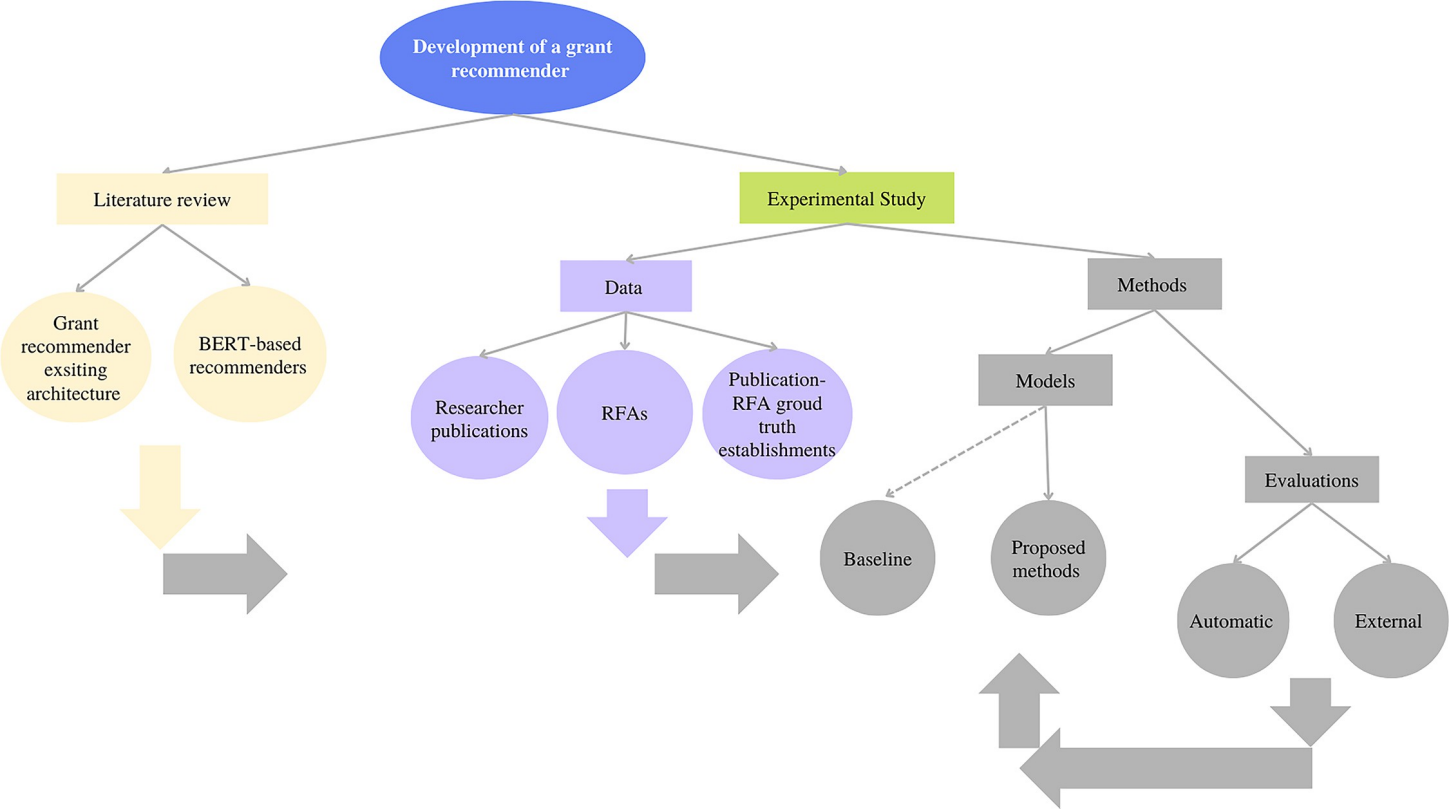
Research methodology overview.

The rest of the article is organized as follows: Related work summarizes literature regarding grant recommendations as well as BERT-based recommenders. An overview of collected grants and publications are provided in the Data section. Methods used for developing the recommendation system and evaluation used in experiments are described in the Methods section. Experimental results and detailed analysis are presented in Results section. Finally, conclusions and discussions, and future directions are discussed in Conclusions and discussions. The overall research methodology is summarized in Fig 1.

## Related work

Literature on grant recommendations is very limited. Kamada et al. [3,4] developed a Japanese grant recommender using keywords and association rules between researchers and grants, and further extended the system with TF-IDF technique. Another system called EILEEN [5] also adopted TF-IDF with Latent Semantic Analysis for topic extractions and used Rocchio Algorithm and Random Forest to predict potential matches of grants and publications.

In addition to our work in [13–17], we were able to locate studies that focus on other academic recommendations and BERT-based recommenders that are related to our research, Patra et al. [16] experimented with information retrieval paradigms (BM25, TF-IDF, etc.) for Gene Expression Omnibus data recommendation to researchers. Zhu et al. [13] utilized graph neural networks to capture intrinsic, complex and changing dependencies among researchers for dynamic collaborator recommendation. Regarding BERT-based systems, Zhu et al. [15] developed a BERT-based recommender to recommend public available papers to researchers. Later Zhu et al. [17] performed a sensitivity analysis on the training class imbalance on BERT-based dataset recommendation system. Bilal et al. [18] used BERT classifier along with three bag-of-words based classifiers to recommend helpful online reviews on Yelp datasets. Jeong et al. [19] combined graph convolution networks with BERT representation of textual data to generate context-aware paper recommendations. Dai et al. [20] introduced a two-stage COVID-19 paper citation recommender by enhancing BERT representation learning in the first stage, and learning effective dense vector of nodes among bibliographic graph through heterogenous deep graph convolutional networks. Hassen et al. [21] compared several popular encoder models including USE, BERT, InferSent, ELMo and SciBERT and found out that solely semantic information from these models did not outperform BM25 for paper recommendations. Yang et al. [22] proposed a semi-supervised research literature and researcher recommendation system using BERT for keywords extraction and Latent Dirichlet Allocation for topic representations.

## Data

The proposed grant recommendation system requires data describing grant announcements and researchers. Grants announcements’ data collected from GRANTS.GOV and the NIH website [23], and researchers’ data created from publications in PubMed. Data collection methods and summaries of data are described next.

### Researcher publications

**Fig 2:**
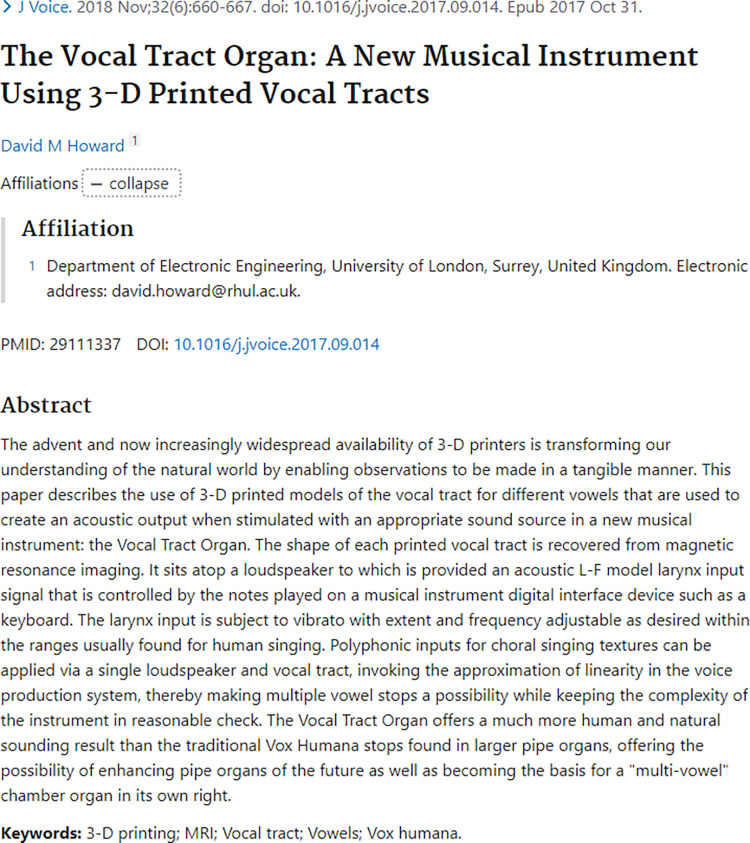
An example of publication details collected.

Published articles downloaded from PubMed database were used to represent researchers. We were particularly interested in articles’ ids, titles, abstracts and dates of publication. A total of 193,592 records were created. An example of PubMed article can be found in Fig 2, and basic word count summary can be found in Table 1.

**Table 1:** Word count summary for researcher publications.

	Title	Abstract
**Mean**	10	121
**Min**	2	0
**50%**	9	126
**Max**	48	844

### Research funding announcements (RFAs)

**Fig 3:**
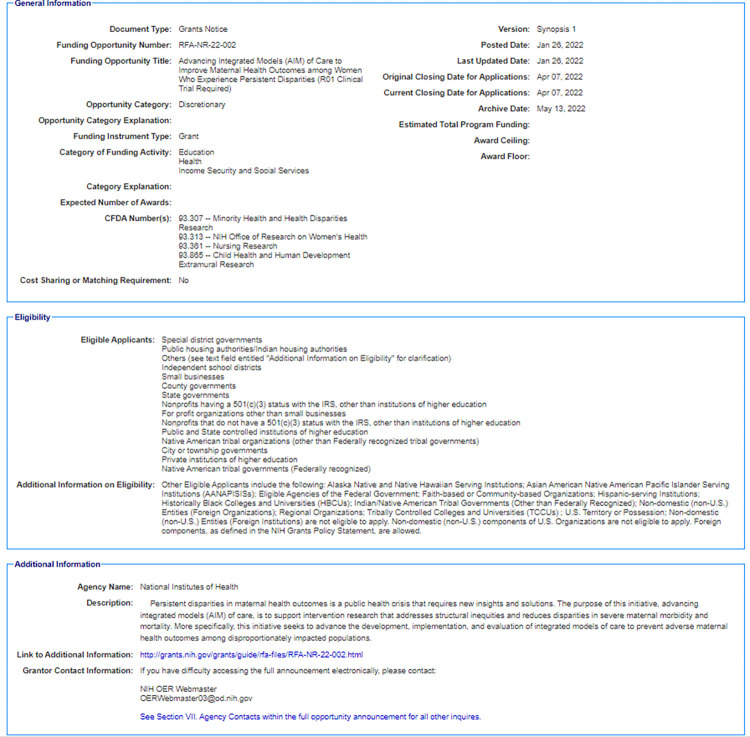
An example of grant details collected.

We crawled GRANTS.GOV because of its comprehensive meta-data and neatly parsed texts. Particularly for our experiments, we were interested in RFA ids, titles as well as descriptions. Since we focused on the biomedical domain, we then kept RFAs that were from NIH only. We had a total of 5,030 grant announcements. An example of a grant’s detail can be found in Fig 3 and basic word count summary can be found in Table 2.

**Table 2:** Word count summary for RFA.

	Title	Description
**Mean**	8	86
**Min**	3	4
**50%**	8	78
**Max**	23	521

### Ground truth establishment

**Fig 4:**
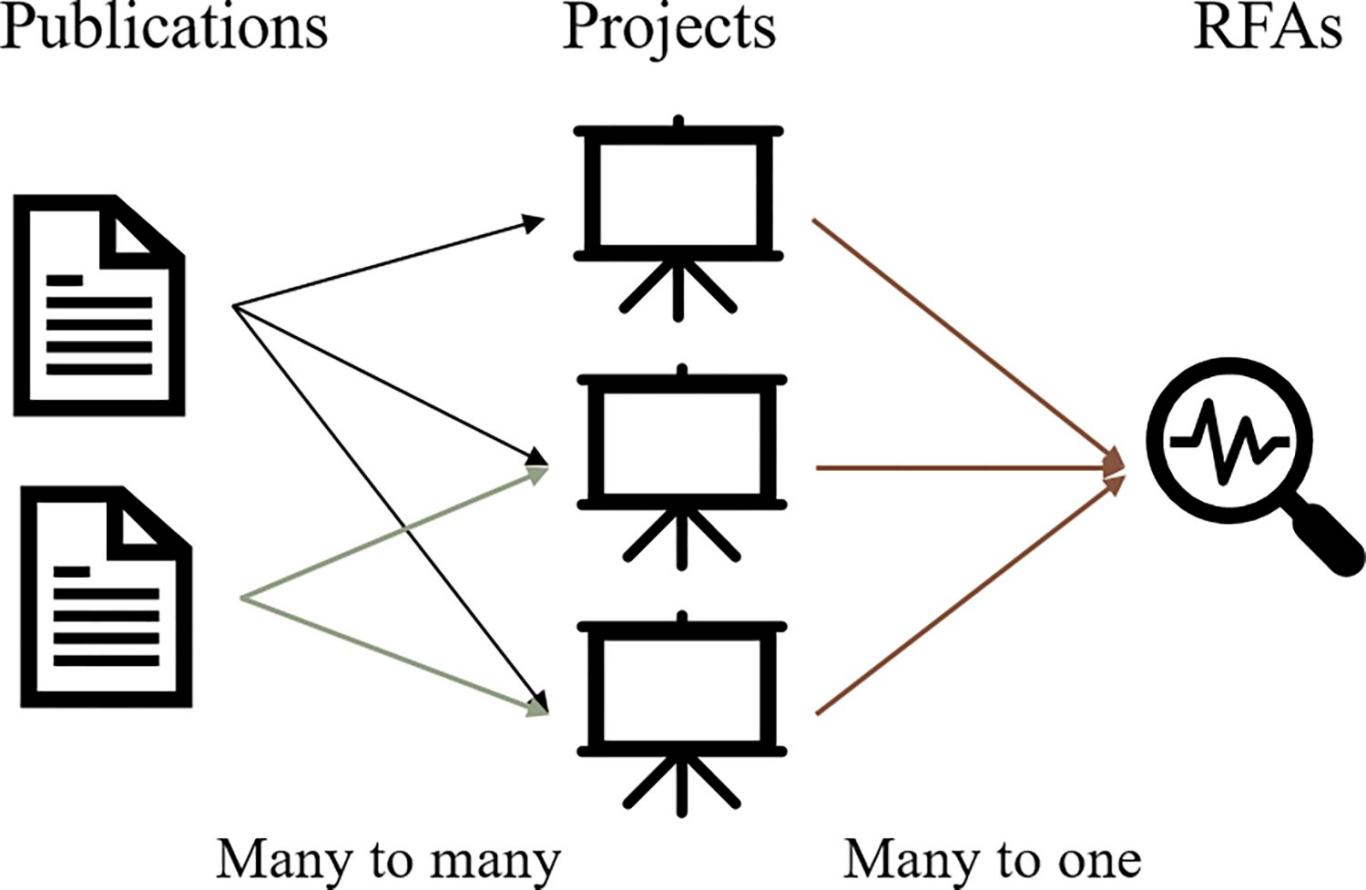
Relations between publications and RFAs through project number.

The relationships between PubMed articles and RFAs were established via NIH’s ExPORTER [24]. It archives relations between publications and project numbers of funded grants, as well as relations between project numbers and corresponding RFAs. Using these two relationships, we could therefore establish the relations between publications and RFAs for evaluation. This relation is then processed into a citation dictionary with each entry recorded as {‘1287764’: [PAR-17-095, PAR-12-298]}, where ‘1287764’ is the PubMed Identifier (PMID) [25], and ‘PAR-17-095’, ‘PAR-12-298’ are the two RFA ids that are associated with this publication. An example of such relationships is provided in Fig 4.

We excluded papers that have too many citations of project numbers (usually survey papers) and limit our final datasets to 193,952 unique papers and 3,678 RFAs.

For training purposes of our proposed method, we need to have both positive (ground truth) and negative (not related) training pairs. Positive training pairs were created out of the existing relations; negative ones were created with random sampling. All possible combinations of publications and RFAs were created first, then positive pairs were excluded from the pool, and finally, an equal number of false pairs were selected. The composite dataset was split on unique publications with ratios 7:1:2 for training, validation and testing, see summaries in Table 3.

**Table 3:** Train, validation and test data.

Splits	# of unique pubs	# of records
Train (7)	135, 766	216, 766
Valid (1)	17,456	28, 056
Test (2)	40, 730	65, 104

## Methods

**Fig 5:**
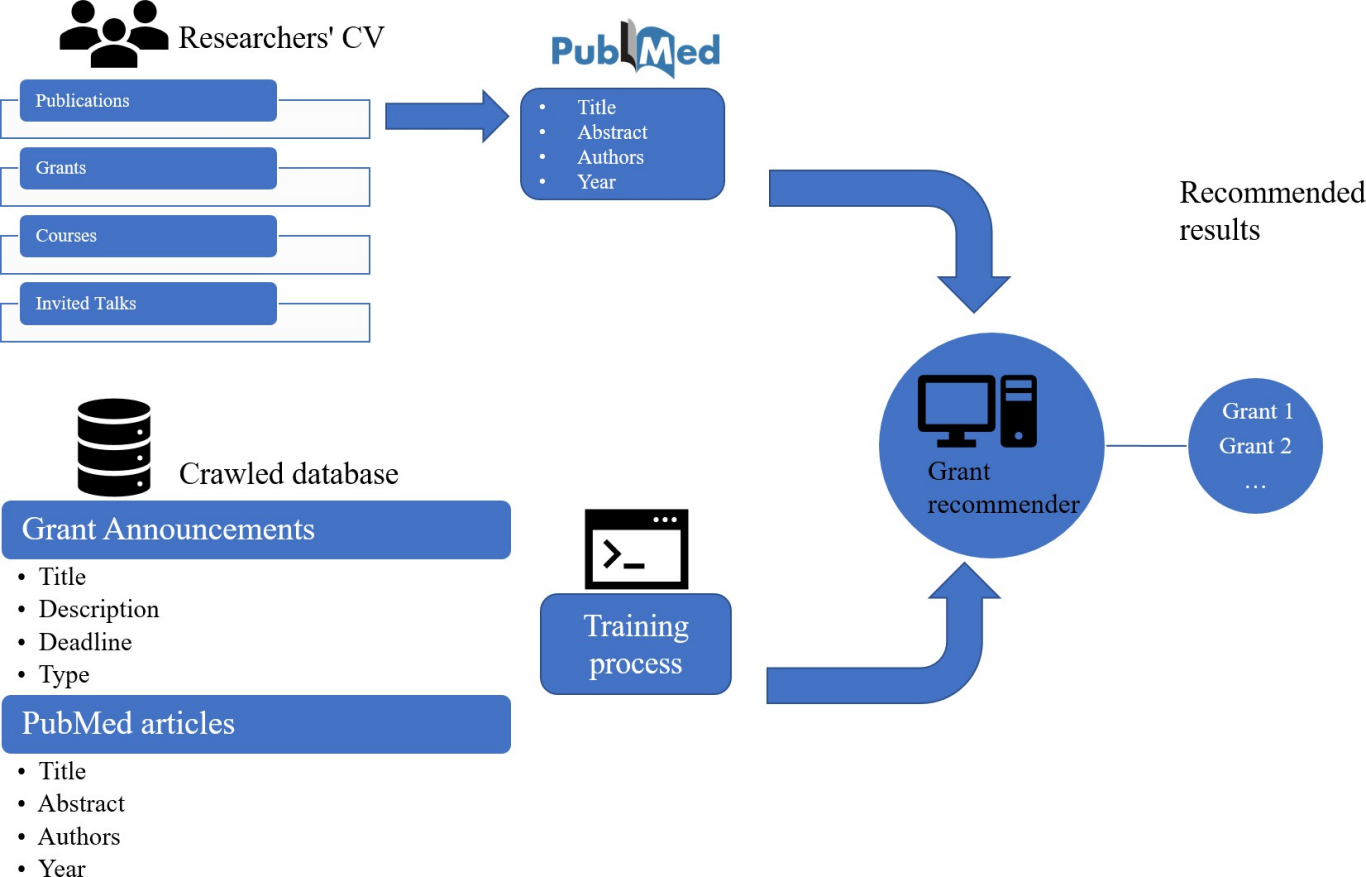
Overview of our grant recommender system architecture.

The overview of the system architecture is outlined in Fig 5. The grant announcement recommendation system developed in this work is part of our Virtual Research Assistant (VRA) project (http://genestudy.org/recommends/#/), a scholarly recommender platform developed at the Department of Biostatistics and Data Science, School of Public Health, The University of Texas Health Science Center at Houston.

There are two main components in our recommender: the offline training component on the bottom, where our model is trained and evaluated against the RFA-publication relationships; and the online test/service component on the top: where researchers/end users submit their information (CVs), and we then use the trained model to provide recommendations. The recommendations are presented in clusters (through clustering on publications and aggregating recommendations per cluster). These aggregated results are then rated by the researchers/end users.

All implementation details could be found at https://github.com/ashraf-yaseen/VRA/tree/master/grants_rec. Below we introduce the main model components and evaluations in details.

### Models

#### Baselines: IR and NB

We built two sets of baseline systems: Information Retrieval (IR)-based and classifier-based. Namely, three IR-based systems utilizing Term Frequency-Inverse Document frequency (TF-IDF), BM25, and doc2vec respectively; the classifier-based system is a Naïve Bayes (NB) classifier combined with the best-performing (on validation data) IR techniques from the three methods.

- TF-IDF: a numerical statistical representation of how important a word is to a document in a collection or corpus [26]. For each vocabulary *V*, the value increases proportionally to the number of times that *V* appears in the document (term frequency, TF) and is offset by the total number of documents that contain *V* (inverse document frequency, IDF). We used TF-IDF implementation from scikit-learn [27].
- BM25: a ranking function that is based on a probabilistic retrieval framework that utilizes adjusted values of TF and IDF and document length [28]. We used BM25 implementation from genism [29].
- doc2vec: an unsupervised neural network that generalizes word2vec and learns continuous distributed vector representations for variable-length pieces of texts [30,31]. We utilized doc2vec implemented in gensim [29].
- NB: A probabilistic classifier based on applying Bayes’ theorem with strong (naïve) independence assumptions between the features given the value of the class variable. It is widely used in document classification tasks (e.g. email spam detections) due to its simplicity and desirable performance. We used the implementation from scikit-learn [27].

For TF-IDF, BM25, and doc2vec, the whole RFAs was used as corpus for retrieval, and publication were used as queries to find the best matching RFAs using cosine-similarity. For NB, we chose the best performing IR techniques on validation data for vector representation and then modeled vectors under the classification labels as the multinomial distribution.

All training parameters can be found in the System parameters section.

#### Proposed method: BERT-based classifier recommender with DPMM and Recency Weight

During initial explorations, we observed that the words in publications and RFAs were not at the same semantic level. For example, more specific words such as ‘clustering genes’, ‘protein analysis’ were present in the publications, whereas the corresponding funding RFAs containing more generic words such as ‘bioinformatics’. Thus, we proposed a classifier recommender using Bidirectional Encoder Representations from Transformer (BERT) to better capture this relationship.

BERT [32] was developed by Google and was pre-trained on 800M-words BooksCorpus [33] and 2500M word English Wikipedia [34] using masked language model and next sentence prediction as the pre-training objectives. It is known for capturing better logical and non-linear information in complex text inputs. It had previously achieved state-of-the-art performance in many classical NLP tasks.

**Fig 6:**
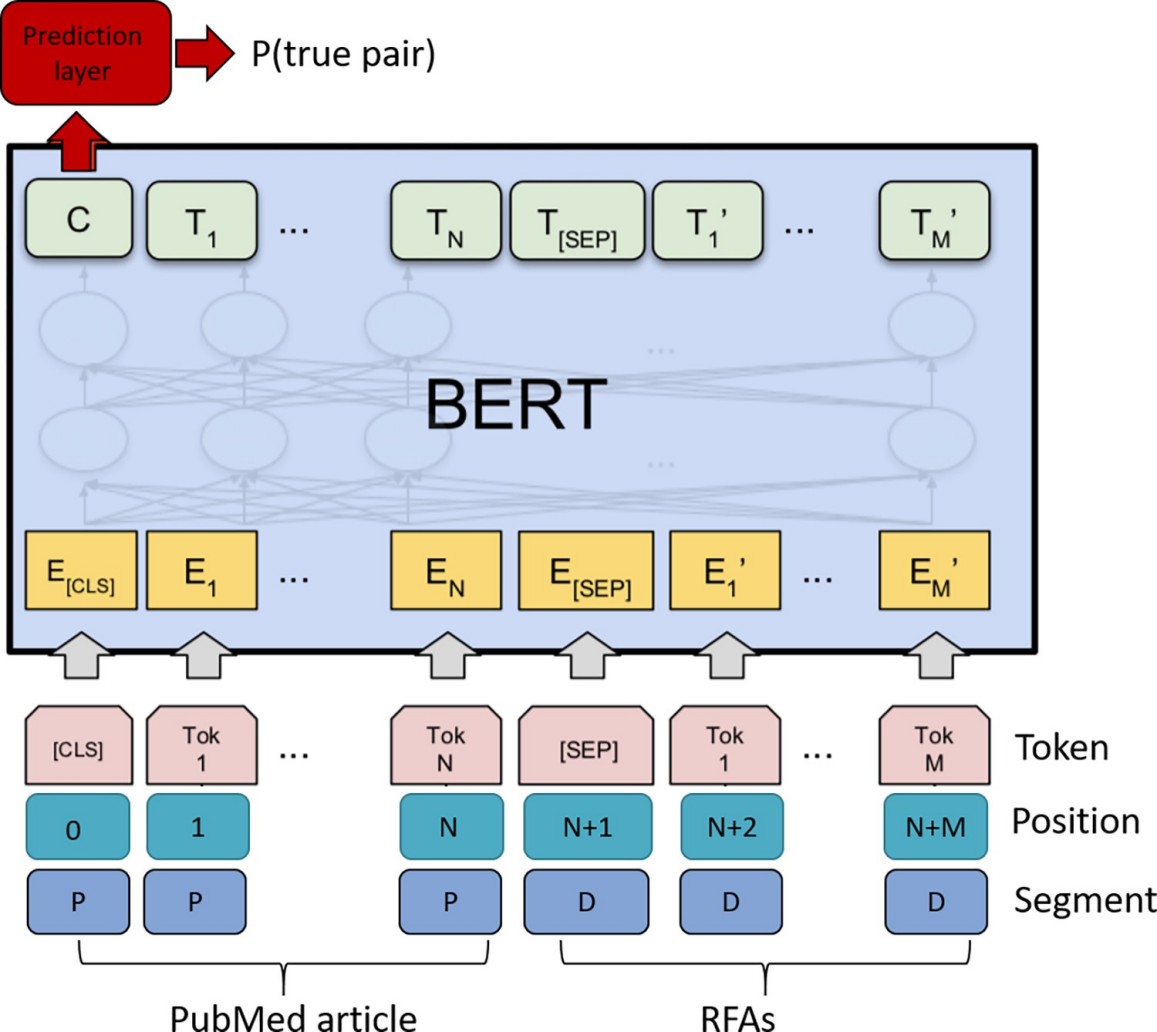
BERT usage in our system.

The goal of the system is to predict whether a particular RFA and a particular publication, and ultimately RFA and a particular researcher, are going to be match. In order to achieve this, we followed a two-stage process. In the first sage, we fine-tuned the base-BERT model using sentence pair classification task, where we defined sentence pair to be “(titles and abstracts of publications, titles and descriptions of the RFAs)”. We truncated both inputs at token size 256 (total 512) with wordPiece tokenizer [35], see Fig 6. The output logits were then converted to probability for aggregating and ranking results. We used Huggingface’s Transformers implementation [36] of base-BERT, and further tuned the model architecture with Ax Bayesian Optimization [37], with final tuned parameters summarized in System parameters.

In the second stage, a particular researcher’s publications are clustered using Dirichlet Process Mixture Model (DPMM), and all RFA-publication results were aggregated based on clusters using Recency Weight, and final recommendations are made per research cluster for each researcher.

DPMM is an iterative non-parametric clustering algorithm that exhibits flexibility in producing varying number of clusters [38] (which suits to the practical needs of our service since each researchers are intrinsically different with varying publication history), scalability, robustness to outliers [39], as well as proven record of success in tasks of document clustering [16,39,40].

Starting with finite mixture model, where each data point is draw from one of the *K* fixed unknown distributions with parameters *θ*_1_,…*θ*_*K*_. Since the number of clusters is unknown, we assume that data point *x*_*n*_ follows a general mixture model in which the parameters are generated from a distribution *G* [41]. The Dirichlet Process (DP) is a stochastic process that generalizes the Dirichlet distribution from being the conjugate prior for a fixed number of categories (multinomial) into the prior for infinitely many categories [38], is characterized by a positive scaling parameter *α* and a base distribution *G*_0_. Assigning a DP prior to *G* in the general mixture model leads to the DPMM [42]. The α value is inversely related to the number of clusters, i.e. decreasing the α parameter in DPMM may increase the number of output clusters. In our case, based on manually observing the clusters and feedback from researchers [16], the α is empirically set as

$$\alpha =\frac{10}{\sqrt{N}}$$

where N is the total number of papers for a researcher.

The complete process is as follow: Publications of a particular researcher (let’s call him/her B) and all available RFAs were made into pairs, and fed into our trained model for prediction of matching probability. Then we took the pairs of ‘positive’ (*Pr*(+)>0.5) predictions and used the probability as the initial matching score (*ms*_*ji*_) of a particular RFA (*j*) to a particular publication (*i*). Then DPMM was introduced to create research clusters (*m*_1_, *m*_2_,…*m*_*B*_) on B’s publications. Once clusters are made, we introduced the Recency Weight *λ*_*i*_ to penalize the initial matching score based on publication year to reflect research interest trend across time:

$$\lambda_{i}=e^{-ct}$$

where *t* is the difference between the year of current experiment and the year of publication. *c* is the decaying factor to decrease the rate proportional to its current value, and for the present study, we kept *c* = 0.05. For rationale, if the publication was published in 1998, the corresponding RFA recommendations are probably of less interest to a research than those for a publication published in 2018. Let’s say this particular publication *i*∈*m*_2_, then we can take the sum of weighted matching scores *ms*_*ji*_ within this cluster *m*_2_ as their final ranking score ${rs}_{jm_{2}}$ for RFA (*j*) for this cluster *m*_2_

$${rs}_{jm_{2}}=\sum_{i=1}^{N_{m_{2}}}\lambda_{i}{ms}_{ji}$$

where $N_{m_{2}}$ is total number of publications in the *m*_2_ cluster. From there, we can take top *K* = 10 final ranking scores’ corresponding RFAs as the recommendations.

#### System parameters

Parameters used during training for baselines vs. our proposed method are all listed in Table 4. We ran several experiments with ranges of values for tuning the parameters of the methods listed in Table 4. The values shown are correspond to best performance. For example, we experiment with a few max_feature options for TF-IDF such as 1000, 2000, and 5000. The performance of the method using max_feature of 2000 slightly outperforms 1000 and no gain in performance when using 5000, so we went with 2000.

**Table 4:** Selective hyperparameters used in baseline vs. our proposed method.

	Methods	Parameters
**Baseline**	TF-IDF	*ngram*_*range*_ = (1,2), *min*_*df*_ = 2, *max*_*features* = 2000
	BM25	*b* = 0.75, *k*_1_ = 1.5
	doc2vec	*dim* = 200, *min*_*count*_ = 2, *epochs* = 50
	NB	*TF*−*IDF same*, *smoothing alpha* = 0.5
**Proposed**	BERT	*Optimzer* = ‘*adam*’, *learning rate* = 2*e*−5, *epochs* = 4
	DPMM	$\alpha =\frac{10}{\sqrt{N}}$
	Recency Weight	*c* = 0.05

### Evaluations

The evaluation was performed in two stages: a) automatic evaluation, where we utilized RFA-publication relationship detailed in Data, Ground truth establishment; b) external evaluation, where experienced researchers were involved in rating recommendations tailored to their profiles. Details are as below.

#### Internal evaluation

This evaluation was developed to verify the effectiveness our proposed method. Metrics were calculated against the ground truth between RFAs and publications that was described in details in Data, Ground truth establishment. Metrics used include Recall@k, Precision@k, Mean Reciprocal Rank (MRR), as well as ROC-AUC. In order to better describe Recall@k and Precision@k, we supplement the confusion matrix as shown in Table 5 below.

**Table 5:** Confusion matrix for recommenders.

	Recommended	Not recommended	Total
**Relevant**	True positive (TP)	False negative (FN)	Total relevant
**Not Relevant**	False positive (FP)	True negative (TN)	Total Not relevant
**Total**	Total recommended	Total Not recommended	Overall Total

- Recall@1: At the k-th retrieved item, this metric measures the proportion of relevant items that are retrieved. We evaluated Recall@1 (R@1).

$$Recall@k=\frac{TP@k}{TP@k+FN}$$

- Precision@1: At the k-th retrieved item, this metric measures the proportion of the retrieved items that are relevant. In our case, we are interested in Precision@1(P@1).

$$Precision@k=\frac{TP@k}{TP@k+FP@k}$$

- Mean reciprocal rank: The Reciprocal Rank (RR) measures the reciprocal of the rank at which the first relevant document was retrieved. RR is 1 if the relevant document was retrieved at rank 1, RR is 0.5 if document is retrieved at rank 2, and so on. When we average retrieved items across the queries *Q*, the measure is called the MRR.

$$MRR=\frac{1}{\left|Q\right|}\sum_{i=1}^{\left|Q\right|}\frac{1}{{rank}_{i}}$$

- ROC-AUC: Area under the ROC curve provides an aggregate measure of discriminating performance across all possible classification thresholds.

For baseline IR methods, we produced the similarity matrix on the test using corpus built on all RFAs, and calculated Recall@1 (R@1), Precision@1 (P@1), and MRR based on the same entries test on classifiers.

For baseline NB, we used the best performing IR from the three previously mentioned methods, and calculated additional ROC-AUC from intermediate results, before we took predicted ‘match’ (1) and aggregated recommendations at publication level for the three metrics mentioned above.

For the proposed method, we calculated the same set of metrics as we did for NB.

#### External evaluation

**Fig 7:**
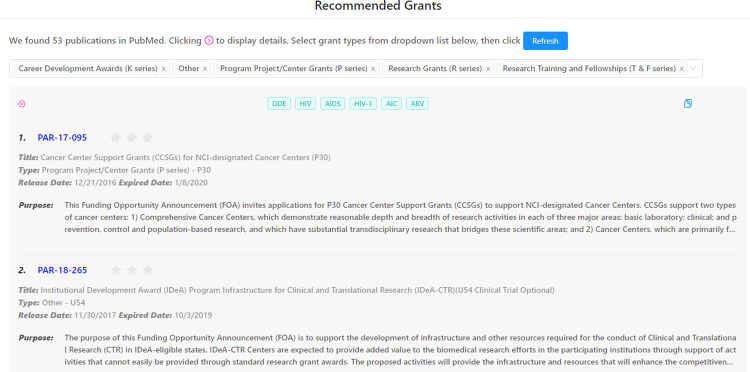
Service platform with evaluation collection.

School of Public Health Departmental professors with a history of grant searches and approvals in the biomedical domain were engaged to evaluate externally our proposed method. We got responses from a total of 10 researchers to participate in the evaluation. After receiving their consent and CVs, researchers’ names were searched in PubMed using a python script for their publications and resultant publications were cross-referenced using their own CVs. Thus, the final total number of papers for reach researcher are different due to their varying years of research history, and our proposed method would produce different number of research clusters, and recommendations corresponding to each cluster. They were asked to rate top 10 recommended grants for each cluster on a scale of 1 to 3 stars based on how satisfied they were with the recommendations, with 3 stars being ‘most satisfied’. We used our grant recommendation platform for collecting results. An example of evaluation platform can be found in Fig 7.

We defined the stars > = 2 as ‘partially relevant’ (P) and 3 stars as ‘strictly relevant’ (S) and calculated Precision@k for these two scenarios and for *k* = 1, 10: P@1(P), P@1(S) and P@10(P), P@10(S), as well as overall average stars.

## Results

The results for automatic evaluations are summarized in Table 6. Since the best performing IR on validation set (results not shown here) was TF-IDF, we used TF-IDF vectorization for NB features.

**Table 6:** Test results for baselines vs. our proposed method.

	Method	R@1	P@1	MRR	ROC-AUC
**Baseline**	TF-IDF	0.69	0.74	0.86	NA
	BM25	0.65	0.69	0.84	NA
	doc2vec	0.65	0.70	0.84	NA
	NB with best vectorizations	0.73	0.87	0.91	0.82
**Proposed**	BERT	**0.81**	**0.87**	**0.93**	**0.98**

We can see that classification-based baseline (NB) outperformed IR baseline, and our proposed method also outperformed classification baseline. Specifically, NB classifier has much worse ROC-AUC comparing to our proposed method, meaning that its overall discriminating power is not on par with the proposed. Since its R@1 is low, NB was not able to identify as much potential matches as our proposed method does, and therefore suffers from coverage problem in its recommendations, even though it has relatively comparable P@1 and MRR.

External evaluation results are summarized in Table 7. 80% of our users gave us average stars > = 2.0 (partially relevant). For our top 1 recommendation, 90% of our users thought they were at least partially relevant (P@1(P)) and 60% of our users thought they were strictly relevant (P@1(S)), across all clusters recommended. For our top 10 recommendations, 70% of our users had a P@10 (P) > 0.9, however all the P@10 (S) were no more than 0.5, indicating that all 3-star percentages for top 10 were not as high as for top 1 hit among users.

**Table 7:** External evaluation of our proposed method.

Users	# of papers	# of clusters	Average stars	P@1 (P)	P@1 (S)	P@10 (P)	P@10 (S)
1	22	4	2.50	1.00	1.00	1.00	0.50
2	4	1	2.40	1.00	1.00	1.00	0.40
3	65	4	2.30	1.00	1.00	1.00	0.30
4	17	5	2.26	1.00	0.60	1.00	0.26
5	39	5	2.16	1.00	0.60	0.96	0.20
6	32	3	2.10	1.00	1.00	0.90	0.20
7	38	7	2.03	1.00	0.43	0.91	0.11
8	65	2	2.00	1.00	1.00	0.50	0.50
9	20	4	1.40	1.00	1.00	0.30	0.11
10	98	8	1.30	0.00	0.00	0.30	0.00

## Conclusions and discussions

To the best of our knowledge, this attempt is the first of its kind to utilize advance, state-of-the-art natural language model, i.e. BERT, to capture intrinsic, non-linear relationship between researchers and grant opportunities dedicated to research grant recommendations. We formulated the problem as a classification task, fine-tuned base-BERT with sentence classification, and paired our core model with DPMM clustering with Recency Weight for final results aggregation for practical applications. Both internal (using RFA-publication relationships) and external evaluation (by users) revealed that our proposed BERT-based system is useful to biomedical researchers.

We think that BERT’s ability to capture intrinsic, non-linear relationship in the publication-RFA pairs greatly contributed to the desirable results compared with baselines. In addition, DPMM allowed us the flexibility to cluster each researcher’s interests differently, and thus provided us a reasonable way to aggregate our recommendations together with our Recency Weights, rendering practicality to the final outputs. However, there are still several limitations regarding our current implementations that call for future actions.

In terms of publication collections for a particular researcher, currently we are using CV cross-references to solve the author name disambiguation [43], i.e., authors with the same name might exist and querying the name in PubMed might sometimes result in publications from other researchers. There are currently a few other approaches that we could possibly explore and compare the effectiveness of performances in the future. One of the most promising one is ORCID [44], which is a persistent digital identifier created especially for the purpose distinguishing researchers with same names. However, many researchers involved in our experiments did not have an associated ORCIDs. By encouraging them to adopt an account, we could ultimately reduce this potential issue. Other methods include rule-based unsupervised [45] as well as supervised approaches [46].

Secondly, since researchers’ publications were crawled from PubMed, there could be a potential discrepancy as publications from most recent conferences or journals might not be timely updated in the database, but they might already appear in researchers’ CVs. Therefore, these publications would not end up as inputs to our system.

In terms of our system architecture, since we need enough amount of publications in the PubMed to begin with, our recommender might not be useful for early-stage researchers. But this problem could be potentially solved by Collaborative Filtering, a technique that utilizes preferences/ratings from other agents, users and data sources [1,47]. This requires a sizeable proportion of user-feedbacks. With our plan to go public with the service in the biomedical domain, we hope to collect useful feedbacks to further improve our system along the way.
